# Glycated haemoglobin (HbA_1c_) and fasting plasma glucose relationships in sea‐level and high‐altitude settings

**DOI:** 10.1111/dme.13335

**Published:** 2017-05-15

**Authors:** J. C. Bazo‐Alvarez, R. Quispe, T. D. Pillay, A. Bernabé‐Ortiz, L. Smeeth, W. Checkley, R. H. Gilman, G. Málaga, J. J. Miranda

**Affiliations:** ^1^ CRONICAS Centre of Excellence in Chronic Diseases Universidad Peruana Cayetano Heredia Lima Peru; ^2^ University College London Medical School London School of Hygiene and Tropical Medicine London UK; ^3^ Faculty of Epidemiology and Population Health London School of Hygiene and Tropical Medicine London UK; ^4^ Division of Pulmonary and Critical Care Johns Hopkins University Baltimore MD USA; ^5^ Área de Investigación y Desarrollo A.B. PRISMA Lima Peru; ^6^ Department of International Health Johns Hopkins Bloomberg School of Public Health Johns Hopkins University Baltimore MD USA; ^7^ Department of Medicine Universidad Peruana Cayetano Heredia Lima Peru

## Abstract

**Aim:**

Higher haemoglobin levels and differences in glucose metabolism have been reported among high‐altitude residents, which may influence the diagnostic performance of HbA_1c_. This study explores the relationship between HbA_1c_ and fasting plasma glucose (FPG) in populations living at sea level and at an altitude of > 3000 m.

**Methods:**

Data from 3613 Peruvian adults without a known diagnosis of diabetes from sea‐level and high‐altitude settings were evaluated. Linear, quadratic and cubic regression models were performed adjusting for potential confounders. Receiver operating characteristic (ROC) curves were constructed and concordance between HbA_1c_ and FPG was assessed using a Kappa index.

**Results:**

At sea level and high altitude, means were 13.5 and 16.7 g/dl (*P* > 0.05) for haemoglobin level; 41 and 40 mmol/mol (5.9% and 5.8%; *P* < 0.01) for HbA_1c_; and 5.8 and 5.1 mmol/l (105 and 91.3 mg/dl; *P* < 0.001) for FPG, respectively. The adjusted relationship between HbA_1c_ and FPG was quadratic at sea level and linear at high altitude. Adjusted models showed that, to predict an HbA_1c_ value of 48 mmol/mol (6.5%), the corresponding mean FPG values at sea level and high altitude were 6.6 and 14.8 mmol/l (120 and 266 mg/dl), respectively. An HbA_1c_ cut‐off of 48 mmol/mol (6.5%) had a sensitivity for high FPG of 87.3% (95% confidence interval (95% CI) 76.5 to 94.4) at sea level and 40.9% (95% CI 20.7 to 63.6) at high altitude.

**Conclusion:**

The relationship between HbA_1c_ and FPG is less clear at high altitude than at sea level. Caution is warranted when using HbA_1c_ to diagnose diabetes mellitus in this setting.

## What's new?

- Haemoglobin levels and differences in glucose metabolism at high altitude may influence the diagnostic performance of testing for diabetes using HbA_1c_.
- We found that the relationship between HbA_1c_ and fasting plasma glucose (FPG) differed markedly between high‐altitude and sea‐level areas.
- The relationship between HbA_1c_ and FPG was quadratic at sea level and linear at high altitude.
- Corresponding FPG values for an HbA_1c_ ≥ 48 mmol/mol (≥ 6.5%) cut‐off point, used for the diagnosis of diabetes, were 6.6 and 14.8 mmol/l (120 and 266 mg/dl) at sea level and high altitude, respectively.
- The sensitivity of HbA_1c_ to detect abnormal FPG was 87.3% at sea level and 40.9% at high altitude. This suggests a limitation in the performance of HbA_1c_ to diagnose diabetes at altitude.

**What's new?**

- Haemoglobin levels and differences in glucose metabolism at high altitude may influence the diagnostic performance of testing for diabetes using HbA_1c_.
- We found that the relationship between HbA_1c_ and fasting plasma glucose (FPG) differed markedly between high‐altitude and sea‐level areas.
- The relationship between HbA_1c_ and FPG was quadratic at sea level and linear at high altitude.
- Corresponding FPG values for an HbA_1c_ ≥ 48 mmol/mol (≥ 6.5%) cut‐off point, used for the diagnosis of diabetes, were 6.6 and 14.8 mmol/l (120 and 266 mg/dl) at sea level and high altitude, respectively.
- The sensitivity of HbA_1c_ to detect abnormal FPG was 87.3% at sea level and 40.9% at high altitude. This suggests a limitation in the performance of HbA_1c_ to diagnose diabetes at altitude.

## Introduction

Nowadays, Type 2 diabetes mellitus is a global epidemic, and the prevalence is far from levelling off. The prevalence of diabetes has almost doubled in the last three decades and the chances of achieving the global target of halting the increase in prevalence by 2025 are < 1% 1. Although originally identified by the presence of glucose in urine, glucose tests for the diagnosis of Type 2 diabetes have been developed over the last century. The oral glucose tolerance test has been used for the diagnosis of Type 2 diabetes over the last three decades. However, this test is laborious for individuals, and thus, has been replaced by fasting plasma glucose (FPG) for use in both clinical settings and epidemiological studies.

HbA_1c_ had been established as the monitoring test of choice to evaluate medium‐term diabetic control 2. Several international societies, including the American Diabetes Association and World Health Organization (WHO) recommend using HbA_1c_ as a diagnostic criterion for Type 2 diabetes in stable haematological circumstances, because it has several advantages over glucose tests, such as low intra‐individual variation and the convenience of taking the test without fasting. However, this recommendation has been criticized because of the observed discordance between HbA_1c_ and glucose tests, and biological variation in certain ethnic groups 3, 4. As such, alternative population‐specific HbA_1c_ cut‐off points for the diagnosis of Type 2 diabetes have recently been proposed 5.

Changes in erythrocytes states, for example, due to folic acid deficiency and renal disease, erythrocyte lifespan and levels of haemoglobin can also influence HbA_1c_ levels 6. One of the mechanisms of adaptation in high‐altitude settings is secondary polycythaemia (increase in haemoglobin levels) 7. A common feature of many Latin American countries, where over the last two decades Type 2 diabetes‐related mortality has been the highest worldwide 8, is a significant proportion of people living at high altitude. Over 30 million people currently reside in the Central American highlands, and in Peru, one third of the population live at altitude 9. Indeed, secondary polycythaemia has been largely reported among Andean natives 10, 11. Additionally, differences in glucose metabolism have been reported among people residing at high altitude 12.

In this study, we aim to explore and compare the relationship between HbA_1c_ and FPG in populations living at high altitude and sea level.

## Methods

### Study settings and participants

We identified eligible individuals from two Peruvian longitudinal population‐based studies: the CRONICAS Cohort Study (*n* = 3601, baseline conducted in 2010–2011), and the rural Ayacucho population of the PERU MIGRANT Study (*n* = 200, baseline conducted in 2007–2008). The CRONICAS Cohort Study aimed to assess the prevalence and incidence of cardiometabolic and pulmonary conditions at four sites: Lima, highly urban, sea level; Tumbes, semi‐urban, sea level; and two high‐altitude locations (3825 m above sea level), rural and urban Puno. All participants were aged 35 years or older and full‐time residents in the area. The PERU MIGRANT Study was designed to investigate differences in cardiovascular disease risk factors between rural‐to‐urban migrant and non‐migrant groups. This study was performed in participants aged 30 years and over from a rural site in Ayacucho, located at 2900–3100 m above sea level, an urban site in Lima, and rural‐to‐urban migrants from Ayacucho currently residing in Lima. In both studies, participants were sex‐ and age‐stratified, a single‐stage random sampling was used, and only one participant per household was enrolled. The studies are described in detail elsewhere 13, 14.

The original pooled dataset had 3801 cases. We excluded 187 individuals with self‐reported diagnosis of diabetes or use of anti‐diabetic medications. In addition, one person was excluded during regression analysis because that individual was an influential point. The final number of people included in this analysis was 3613.

Participants were classified into two geography‐based categories: (1) sea‐level population (those from Lima and Tumbes), and (2) high‐altitude population (those from Ayacucho and Puno).

### Study variables

We evaluated clinical variables, including BMI, hypertension (systolic blood pressure ≥ 140 mmHg or diastolic blood pressure ≥ 90 mmHg or current use of antihypertensive medications), and current smoking status (self‐report of having smoked at least one cigarette in the last 30 days). We also explored sociodemographic variables, such as wealth index based on asset possessions 15 and educational level (primary or less, secondary and higher).

Besides FPG (mmol/l) and HbA_1c_ (mmol/mol; %), additional laboratory variables included lipid measurements (total cholesterol, triglycerides, HDL‐C and Friedewald‐estimated LDL‐C, in mg/dl), and haematological parameters, such as total haemoglobin (g/dl), mean corpuscular volume (fl/red blood cell), mean corpuscular haemoglobin (pg/cell) and mean corpuscular haemoglobin concentration (g/dl). HbA_1c_ was measured using high‐performance liquid chromatography (HPLC, D10‐BIORAD, Germany), which is traceable to the Diabetes Control and Complications Trials reference study as certified by the National Glycohemoglobin Standardization Program.

### Statistical analysis

For descriptive purposes, study variables were compared between sea‐level and high‐altitude settings using analysis of variance, chi‐squared or Fisher's exact tests. Linear, quadratic and cubic regression models were performed to assess the relationship between HbA_1c_ and FPG, crude and adjusted by age, sex, education, wealth, BMI and total haemoglobin. Models were performed separately for each sea‐level and high‐altitude subgroup.

Beta coefficients and 95% confidence intervals (95% CI) were calculated for FPG, squared‐FPG and/or cubic‐FPG. Maximum likelihood optimization (Newton–Raphson) and robust variance estimations 16 were used in these models to compensate for heteroscedasticity and non‐normality. Information from Wald's test and Bayesian information criteria helped select the best models.

We evaluated diagnostic performance for diabetes and prediabetes in the sea‐level and high‐altitude subgroups using FPG as the gold standard. We used the cut‐off points recommended by the American Diabetes Association for HbA_1c_ (normal < 39 mmol/mol, < 5.7%; prediabetes 39 to < 48 mmol/mol, 5.7 to < 6.5%; diabetes ≥ 48 mmol/mol, ≥ 6.5%) and FPG (normal < 5.6 mmol/l; prediabetes 5.6–6.9 mmol/l; diabetes ≥ 7.0 mmol/l). We evaluated sensitivity, specificity, positive predictive value, negative predictive value and likelihood ratios of HbA_1c_, and receiver operating characteristic (ROC) curves.

Finally, we compared concordance between diagnosis by HbA_1c_ and FPG using a Kappa index. All analyses were conducted using Stata/IC v. 12 (Stata Corp, College Station, TX, USA). Ethical approval was obtained for the original studies.

## Results

In total, 3613 individuals were included in the analysis: Lima, *n* = 1036; Tumbes, *n* = 963; Ayacucho, *n* = 200; and Puno, *n* = 1414. Haemoglobin levels were significantly lower in individuals at sea level (13.5 ± 1.4 g/dl) than those at high altitude (16.7 ±1.9 g/dl) (*P* < 0.001). Mean HbA_1c_ was 41 mmol/mol (5.9 ± 0.88%) at sea level, and 40 mmol/mol (5.8 ± 0.48%) at high altitude. Individuals at sea level had higher mean FPG (5.3 ± 1.4 mmol/l) compared with those from high altitude (4.9 ± 0.9 mmol/l) (*P* < 0.001). The cardiovascular risk factor profile, in terms of adiposity, lipid markers, hypertension and smoking status, was poorer among those living at sea level. Diabetes, defined both by HbA_1c_ and FPG, was more prevalent at sea level than high altitude. In both high‐altitude and sea‐level settings, the estimates of HbA_1c_‐defined diabetes were three times higher than those based on FPG. We had haematological parameters from one site at high altitude (Ayacucho, *n* = 167). Levels of mean haematocrit were 48.5 ± 4.1%; mean corpuscular volume was 94.9 ± 4.9 fl/red blood cell; mean corpuscular haemoglobin was 31.2 ± 1.7 pg/cell; and mean corpuscular haemoglobin concentration was 32.8 ± 1.2 g/dl (Table 1).

**Table 1:** Characteristics of study participants at sea‐level and high‐altitude settings

Variable	Total (*n* = 3613)	Sea level (*n* = 1999)	High altitude (*n* = 1614)	*P* a
Sociodemographic				
Age (mean ± sd)	3611	55.2 ± 12.7	55.0 ± 13.0	0.77
Male, *n* (%)	3610	986 (49.3)	770 (47.8)	0.36
Wealth index (mean ± sd)	3613	251.9 ± 153.8	167.9 ± 161.8	< 0.001
Education				
Primary or less, *n* (%)	1709	971 (48.6)	738 (45.7)	< 0.001
Secondary, *n* (%)	1137	707 (35.4)	430 (26.7)	
Higher, *n* (%)	764	319 (16.0)	445 (27.6)	
Cardiovascular risk factors				
BMI (kg/m^2^, mean ± sd)	3248	28.3 ± 4.6	25.9 ± 4.2	< 0.001
Waist circumference, cm (mean ± sd)	3243	93.2 ± 10.4	86.6 ± 12.1	< 0.001
Total cholesterol, mg/dL (mean ± sd)	2947	202.4 ± 38.7	194.7 ± 40.8	< 0.001
Triglycerides, mg/dL [median (IQR)]	3147	139 (97)	125 (83)	< 0.001
HDL‐C, mg/dL (mean ± sd)	2947	40.9 ± 11.5	43.0 ± 11.3	< 0.001
LDL‐C, mg/dL (mean ± sd)^b^	200	–	85.7 ± 27.1	
Hypertension, *n* (%)	3047	291 (15.0)	106 (9.6)	< 0.001
Current smoker, *n* (%)	3610	268 (13.4)	130 (8.1)	< 0.001
Haematological variables				
Haemoglobin, g/dL (mean ± sd)	3146	13.5 ± 1.4	16.7 ± 1.9	< 0.001
Mean corpuscular volume, fl/red blood cell^b^	167	–	94.9 ± 4.9	
Mean corpuscular haemoglobin, pg/cell^b^	167	–	31.2 ± 1.7	
Mean corpuscular haemoglobin concentration, g/dl^b^	167	–	32.8 ± 1.2	
Diabetes‐related markers				
HbA_1c_ (mean mmol/mol)	3146	41	40	0.10
HbA_1c_ (mean % ± sd)		5.9 ± 0.88	5.8 ± 0.48	
Fasting plasma glucose (mean mmol/l ± sd)	3146	5.3 ± 1.4	4.9 ± 0.9	< 0.001
Diabetes diagnosed by HbA_1c_ c				
Normal, *n* (%)	1227	789 (40.9)	438 (36.0)	< 0.001
Prediabetes, *n* (%)	1687	978 (50.6)	709 (58.3)	
Diabetes, *n* (%)	232	163 (8.5)	69 (5.7)	
Diabetes diagnosed by FPG^d^				
Normal, *n* (%)^a^	2493	1433 (74.3)	1060 (87.2)	< 0.001
Prediabetes, *n* (%)^a^	568	434 (22.5)	134 (11.0)	
Diabetes, *n* (%)^a^	85	63 (3.3)	22 (1.8)	

a ANOVA one‐way for mean differences; Kruskal–Wallis or median differences; chi square for distribution differences.

b Only available for Ayacucho.

c Prediabetes and diabetes were diagnosed using the American Diabetes Association recommended HbA_1c_ cut‐off point: diabetes, HbA_1c_ ≥ 48 mmol/mol (≥ 6.5%); prediabetes, ≥ 48 mmol/mol (6.5%) > HbA_1c_ ≥ 39 mmol/mol (≥ 5.7%); normal, HbA_1c_ < 39 mmol/mol (< 5.7%).

d Prediabetes and diabetes were diagnosed using the American Diabetes Association recommended FPG cut‐off point: diabetes, FPG ≥ 7.0 mmol/l; prediabetes, 7.0 mmol/l > FPG ≥ 5.6 mmol/l; normal: FPG < 5.6 mmol/l.

FPG, fasting plasma glucose.

**Figure 1:**
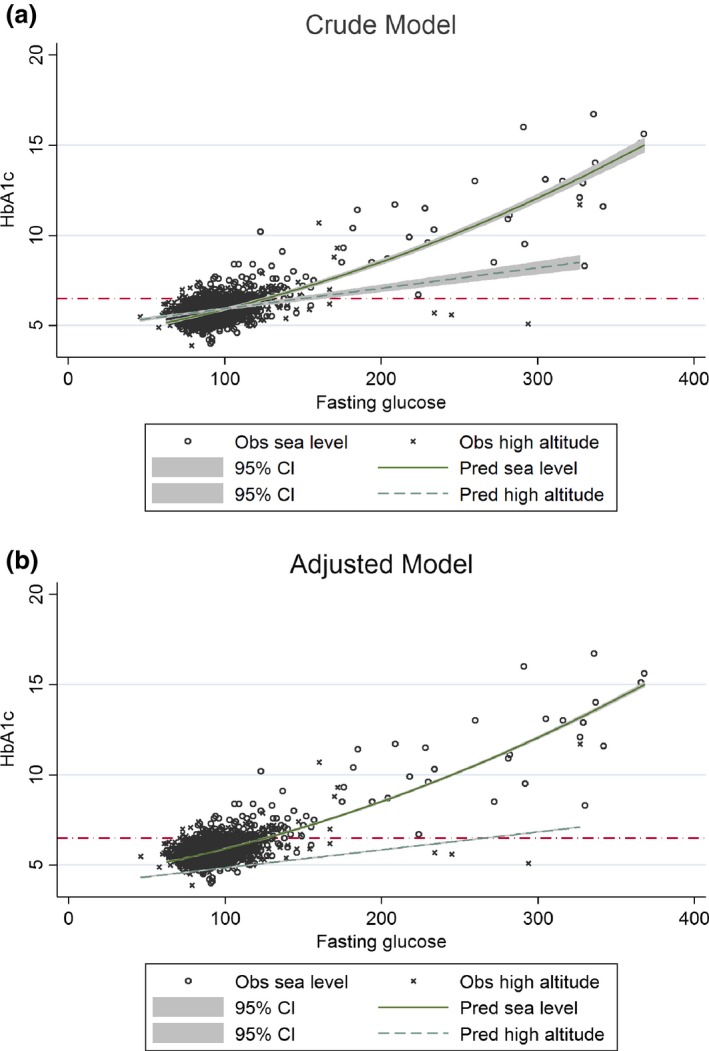
Graphical representation of the quadratic model (sea level) and linear model (high altitude) for HbA_1c_ (dependent variable) and fasting plasma glucose (independent variable), crude and adjusted by age, sex, education, wealth, BMI and total haemoglobin levels. After comparison of linear, quadratic and cubic models of the relationship between HbA_1c_ and fasting plasma glucose, a quadratic adjusted model was selected as the best for people at sea level, and a linear adjusted model was selected as the best for people at high altitude (Table S1). The red line was established at an HbA_1c_ value of 48 mmol/mol (6.5%) to represent the current recommended diagnostic cut‐point for diabetes 17.

**Figure 2:**
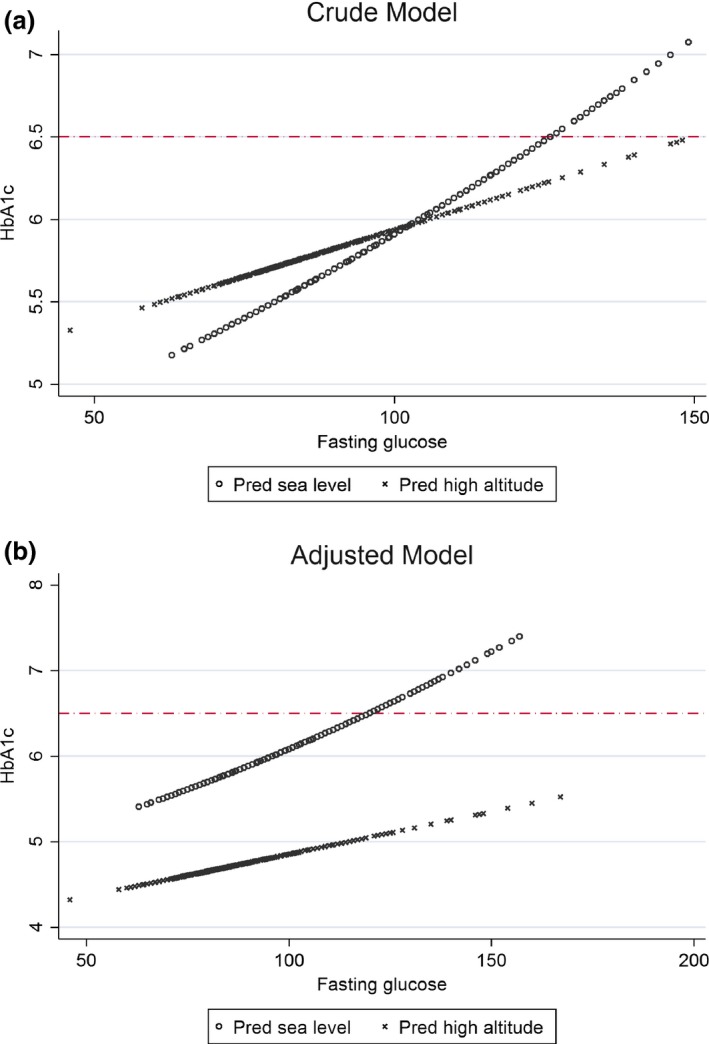
Amplification of the zone near to an HbA1c value of 48 mmol/mol (6.5%) in the graphical representation of the quadratic model (sea level) and linear model (high altitude) for HbA_1c_ (dependent variable) and fasting plasma glucose (independent variable) both crude and adjusted by age, sex, education, wealth, BMI and total haemoglobin. The red line was established at a HbA_1c_ value of 48 mmol/mol (6.5%) to indicate the standard diagnostic cut‐point for diabetes 17.

In both crude and adjusted models, we found differences between predictions of HbA_1c_ by FPG at sea level and high altitude (Figs 1 and 2). Whereas HbA_1c_ and FPG showed a non‐linear, quadratic relationship at sea level, we found a linear association at high altitude (Table S1). Differences in relationship patterns and intercept values (3.9 for high altitude, 4.6 for sea level) display notable differences in the shape of each curve (Fig. 1). This effect has a repercussion on values for diagnosis: to predict an HbA_1c_ value of 48 mmol/mol (6.5%), mean FPG values of 6.6 and 14.8 mmol/l were needed at sea level and high altitude, respectively.

Among those with diabetes, at sea level, the number of individuals diagnosed by HbA_1c_ was 13.5 times greater than diagnosed by FPG only (108 vs. 8), and this relationship was 4.6 times greater at high altitude (60 vs. 13). Individuals diagnosed by HbA_1c_ only were older, but metabolically healthier at high altitude than at sea level. Similar results were found among individuals diagnosed by FPG, and by the combination of HbA_1c_ and FPG (Table 2).

**Table 2:** Clinical characteristics of individuals with diagnosis of diabetes by different criteria^a^

	Overall			Sea‐level population			High‐altitude population		
Variable	HbA_1c_ only	HbA_1c_ and FPG	FPG only	HbA_1c_ only	HbA_1c_ and FPG	FPG only	HbA_1c_ only	HbA_1c_ and FPG	FPG only
*n* b	168	64	21	108	55	8	60	9	13
Age (mean ± sd)	60.4 ± 12.8	57.2 ± 10.5	58.4 ± 12.3	59.1 ± 12.9	57.0 ± 10.3	53.8 ± 14.1	62.7 ± 12.2	58.7 ± 12.0	61.3 ± 10.6
Male, *n* (%)	59 (35.1)	26 (40.6)	12 (57.1)	**42 (38.9)**	**23 (41.8)**	**5 (62.5)**	**17 (28.3)**	**3 (33.3)**	**7 (53.9)**
BMI (kg/m^2^)	29.6 ± 6.0	30.6 ± 5.1	28.5 ± 6.1	**30.7 ± 5.8**	30.9 ± 5.0	**33.0 ± 7.8**	**27.6 ± 5.9**	28.9 ± 5.3	**25.7 ± 2.4**
Waist circumference, cm (mean ± sd)	95.1 ± 13.4	99.0 ± 9.2	94.4 ± 15.0	**98.3 ± 11.0**	99.7 ± 11.3	**104.9 ± 17.5**	**89.3 ± 15.4**	95.0 ± 13.5	**87.9 ± 8.9**
Total cholesterol, mg/dL (mean ± sd)	208.8 ± 42.9	218.0 ± 39.4	220.0 ± 42.3	211.8 ± 39.4	217.7 ± 40.8	236.6 ± 43.2	201.9 ± 49.7	219.5 ± 29.9	209.6 ± 39.9
Triglycerides, mg/dL [median (IQR)]	149 (101)	184 (100)	112 (81)	148 (97)	183 (108)	138 (66)	149 (105)	185 (96)	111 (84)
HDL‐C, mg/dL (mean ± sd)	39.0 ± 10.3	38.6 ± 10.6	50.1 ± 15.3	38.7 ± 9.8	38.5 ± 11.1	48.1 ± 17.8	39.8 ± 11.6	39.4 ± 7.8	51.3 ± 14.2
Hypertension, *n* (%)	34 (22.1)	20 (31.8)	2 (9.5)	28 (25.9)	20 (36.4)	2 (25.0)	6 (13.0)	0 (0)	0 (0)
Current smoker, *n* (%)	23 (13.7)	11 (17.2)	4 (19.1)	16 (14.8)	10 (18.2)	2 (25.0)	7 (11.7)	1 (11.1)	2 (15.4)
Haemoglobin, g/dL (mean ± sd)	14.5 ± 2.6	14.3 ± 2.2	16.0 ± 2.0	**13.2 ± 1.7**	**13.6 ± 1.1**	**14.3 ± 0.98**	**16.9 ± 2.3**	**18.5 ± 2.5**	**17.0 ± 1.8**

a Diagnosed by HbA_1c_ only: HbA1c ≥ 48 mmol/mol (≥ 6.5%) and FPG < 7.0 mmol/l; diagnosed by FPG only: HbA_1c_ < 48 mmol/mol (< 6.5%) and FPG ≥ 7.0 mmol/l; diagnosed by both: HbA_1c_ ≥ 48 mmol/mol (≥ 6.5%) and FPG ≥ 7.0 mmol/l.

b Of a total 3613 people in the study, we excluded those without diabetes (*n* = 2892) and those without complete data to evaluate diabetes status (HbA_1c_ and FPG, *n* = 468), therefore data from *n* = 253 people is included.

Entries in bold represent *P* < 0.05 in comparisons between sea‐level and high‐altitude populations. For categorical variables (%) we used Fisher's exact test (2 × 3 cross table). For continuous variables (means) we used one‐way ANOVA, comparing each criterion by altitude (separately). For non‐normal variables (medians) we used Kruskal–Wallis test, comparing each criterion by altitude (separately).

FPG, fasting plasma glucose.

Using HbA_1c_ instead of FPG to diagnose diabetes increased the number of cases by 159% at sea level and 215% at high altitude. Finally, when evaluating the agreement between diagnosis of diabetes and prediabetes by HbA_1c_ and FPG, we found poor agreement at sea level (Kappa = 0.19) and at high altitude (Kappa = 0.04) (Table 3).

**Table 3:** Concordance of diabetes and prediabetes diagnostics at sea level and high altitude settings considering HbA1c or FPG standard cut‐points

Test		Sea‐level population				High‐altitude population			
		HbA_1c_				HbA_1c_			
		Normal	Prediabetes	Diabetes	Total	Normal	Prediabetes	Diabetes	Total
Fasting plasma glucose	Normal	692	691	50	1433	402	619	38	1059
	Prediabetes	95	281	58	434	32	80	22	134
	Diabetes	2	6	55	63	4	9	9	22
	Total^a^	789	978	163	1930	438	708	69	1215

a Of a total of 3613 people in the study, we excluded those without complete data to evaluate diabetes status (HbA_1c_ and FPG, *n* = 468), therefore, data from *n* = 3145 people are included.

Diagnostic criteria for HbA_1c_: diabetes, HbA_1c_ ≥ 48 mmol/mol (≥ 6.5%); prediabetes, ≥ 48 mmol/mol (6.5%) > HbA_1c_ ≥ 39 mmol/mol (≥ 5.7%); normal: HbA_1c_ < 39 mmol/mol (< 5.7%).

Diagnostic criteria for FPG: diabetes, FPG ≥ 7.0 mmol/l; prediabetes, 7.0 > FPG ≥ 5.6 mmol/l; normal, FPG < 5.6 mmol/l.

Concordance at sea‐level settings: kappa = 0.19, expected agreement = 42.0%; agreement = 53.3%.

Concordance at high‐altitude settings: kappa = 0.04, expected agreement = 38.0%; agreement = 40.4%.

FPG, fasting plasma glucose.

The sensitivity of an HbA_1c_ cut‐off value 48 mmol/mol (6.5%) for diabetes diagnoses, using FPG as a gold standard, was much higher in the sea‐level groups (87.3%) than in the high‐altitude groups (40.9%), with specificities of 94.2% and 95.0%, respectively. Positive likelihood ratios were 15.1 and 8.1, respectively. Sensitivities for diagnosis of prediabetes were similar, 74.7% and 71.4% in the sea‐level and high‐altitude groups, respectively (Table 4). ROC areas for sea level (0.95) and high altitude (0.74) were significantly different (chi^2^(1) = 9, *P* < 0.01) using HbA_1c_ standard cut‐points and FPG as the gold standard of diabetes (Fig. S1). ROC areas for sea level (0.68) and high altitude (0.57) were also significantly different (chi^2^(1) = 12, *P* < 0.001) using HbA_1c_ standard cut‐points and FPG as the gold standard of prediabetes (Fig. S2).

**Table 4:** Diagnostic test characteristics for HbA_1c_ standard cut‐points using FPG as the gold standard

		Sea‐level population (*n* = 1930)^a^		High‐altitude population (*n* = 1215)^a^	
	Test	%	95% CI	%	95% CI
Diabetes, HbA_1c_ ≥ 48 mmol/mol (≥ 6.5%)	Sensitivity	87.3	(76.5–94.4)	40.9	(20.7–63.6)
	Specificity	94.2	(93.1–95.2)	95.0	(93.6–96.1)
	PPV	51.2	(46.1–56.3)^b^	25.3	(16.2–37.2)^b^
	NPV	99.1	(98.2–99.5)^b^	97.5	(96.5–98.2)^b^
	LR+	15.1	(12.3–18.5)	8.1	(4.7–14.2)
	LR−	0.14	(0.07–0.26)	0.62	(0.44–0.88)
Prediabetes, HbA_1c_ ≥ 39 mmol/mol (≥ 5.7%) and < 48 mmol/mol (< 6.5%)	Sensitivity	74.7	(70.0–79.0)	71.4	(62.1–79.6)
	Specificity	50	(47.4–52.7)	39.4	(36.4–42.4)
	PPV	32.1	(30.4–33.8)^b^	13.8	(12.4–15.4)^b^
	NPV	86.2	(83.9–88.3)^b^	91.0	(88.2–93.2)^b^
	LR+	1.5	(1.4–1.6)	1.2	(1.1–1.3)
	LR−	0.51	(0.42–0.61)	0.73	(0.54–0.98)

a Of a total 3613 people in the study, we excluded those without complete data to evaluate diabetes status (HbA_1c_ and FPG, *n* = 468), therefore data from *n* = 3145 people are included.

b Values and confidence intervals are based on likelihood ratios, using prevalence estimated in this study (using explained FPG cut‐off points). Diabetes sea level = 6.5%; diabetes high altitude = 4%; prediabetes sea level = 24%; prediabetes high altitude = 12%.

The gold standard for diabetes is defined as FPG ≥ 7.0 mmol/l and for prediabetes 7.0 mmol/l > FPG ≥ 5.6 mmol/l.

95% CI, 95% confidence interval; FPG, fasting plasma glucose; PPV, positive predictive value; NPV, negative predictive value; LR+, positive likelihood ratio; LR−, negative likelihood ratio.

The prevalence of diabetes is higher when HbA_1c_ is used (≥ 48 mmol/mol; ≥ 6.5%) rather than FPG (≥ 7.0 mmol/l) for sea‐level populations (diabetes prevalence of 12.3% with HbA_1c_ and 6.5% with FPG) and high‐altitude populations (diabetes prevalence of 7.9% with HbA_1c_ and 3.8% with FPG) (Table S2).

## Discussion

In this study, we found that the relationship between HbA_1c_ and FPG differed markedly between populations living at high altitude and sea level. Using current recommended HbA_1c_ cut‐off points for the diagnosis of diabetes (≥ 48 mmol/mol, ≥ 6.5%), our models showed a discrepancy of up to 8.2 mmol/l units of FPG. In other words, corresponding FPG values for such HbA_1c_ cut‐off point were 6.6 and 14.8 mmol/l at sea level and high altitude, respectively. This translated into major discrepancies in diagnostic performance, as shown by differences in the sensitivity of HbA_1c_ at sea level (89%) compared with at high altitude (41%). In terms of new cases of diabetes, greater discordance was observed in high‐altitude settings, which was confirmed by the poor agreement found. Taken together, our findings show that high altitude is another setting in which HbA_1c_ might not be appropriate when used as a diagnostic tool for Type 2 diabetes.

Discordance between FPG and HbA_1c_ has been reported in American, European and Asian populations, as well as in older and female individuals. However, this is the first study reporting discordance in Andean populations. Differences in the glycation process, of genetic or adaptive origin, have been shown to play a significant role in inter‐individual variance by causing abnormally high or low levels of HbA_1c_ for a given plasma glucose level 18. However, other physiological or environmental pathways may contribute to discordance observed between FPG and HbA_1c_ in our study settings.

We observed that FPG was, on average, 0.4 mmol/l higher at sea‐level sites than at high‐altitude sites, yet mean HbA_1c_ was similar in both study groups. Glucose metabolism has been shown to differ at altitude; for instance, an association between polycythaemia and glucose intolerance has previously been described in an Andean population 12. A study in rats showed that exposure to hypobaric hypoxia is associated with reduced insulin release due to inhibition of corticotrophin‐releasing hormone 19. This has also been replicated in clinical research; a recent publication by our group found that a 5% decrease in oxyhaemoglobin saturation was strongly associated with a HbA_1c_ value ≥ 48 mmol/mol (≥ 6.5%) (JC Bazo‐Alvarez, R Quispe, TD Pillay, A Bernabé‐Ortiz, L Smeeth, W Checkley, RH Gilman, G Málaga, JJ Miranda, personal communication). It is possible that these processes of relative intolerance lead to a serum glucose level that is not represented by a FPG test obtained in a fasting state, but is identified by HbA_1c_.

We hypothesize that the discrepancy observed in our study might be partially explained by an increase in haemoglobin production. An erythropoietin‐driven increase in haemoglobin production is the most important mechanism of adaptation and acclimatization seen at altitude, especially in the Andes 20, 21. This observation was further confirmed in our study, because we found significantly higher mean haemoglobin levels at high altitude than at sea level. Increased erythropoiesis due to other causes, such as intravenous iron or erythropoietin‐stimulating agents, has also been shown to influence HbA_1c_ levels 22, 23, 24. In high‐altitude native populations, the utilization of iron appears to be 25% greater than in people from sea‐level settings 25, and higher HbA_1c_ values have been reported in individuals with iron deficiency 26, 27. Haemoglobin levels may affect the extent of glycation, however, the effect of altitude on lifespan remains unclear 28. Our haematological data were limited and thorough evaluation of haematological parameters in relationship to glucose and other metabolic markers will add to this understanding. Another potential mechanism to explain our observations might be related to haemoglobin glycation itself at altitude, although the literature is limited in this field. Indeed, many of the mechanisms presented deserve to be fully studied in high‐altitude settings.

Mounting evidence from observational and controlled clinical trials has demonstrated a strong association between HbA_1c_ levels and retinopathy and other microvascular complications of diabetes. Moreover, HbA_1c_ is also associated with increased risk of cardiovascular disease, even in individuals without diabetes 29. As such, the role of HbA_1c_ as a key biomarker is undeniable. Yet, the discordance between FPG and HbA_1c_ at altitude observed in our study, particularly in high‐altitude settings, merits further and deeper scrutiny because the number of people classified as having diabetes would treble if HbA_1c_ was used as a diagnosis tool. This discrepancy between HbA_1c_ and FPG has recently been highlighted in a global data‐pooling study signalling difficulties for monitoring of diabetes targets at a policy level 30. Consequently, many individuals residing at high altitude, who were shown to have a more favourable cardiometabolic risk profile than those residing at sea level, would initiate glucose‐lowering medications and be exposed to the unnecessary harm associated with such treatments. Given the large populations living at high altitude and the rising prevalence of diabetes worldwide, especially in the southern hemisphere, inappropriate prescription of anti‐diabetic medications might lead to inefficient public health policies in countries with limited economic resources.

This study has benefited from leveraging data from well‐defined population‐based studies and relatively large sample sizes. Peru has a particular geographical distribution characterized by a large variety of climates and altitudes. The CRONICAS Cohort Study and the PERU MIGRANT Study cohorts are unique in that they have a relatively large proportion of individuals living at high altitude, > 3000 m above sea level, where increases in haemoglobin levels are mostly observed. Despite this, our study did not have data on oral glucose tolerance test, the gold standard test used for diabetes research, multiple FPG readings over time to more accurately represent glucose levels in people with diabetes, or a detailed evaluation of all haematological markers in all sites. The cross‐sectional approach of this study precludes the ascertainment of causal relationships; therefore, longitudinal studies are better placed to explore the long‐term consequences of the discordant patterns reported, particularly in terms of progression of diabetes‐related complications. Prospective evaluations are also required to evaluate clinical and economic consequences that may result from modification of current diagnostic criteria.

## Conclusions

These findings provide unique evidence that the relationship between HbA_1c_ and FPG differs considerably between sea‐level and high‐altitude settings. Our models show that an HbA_1c_ of 48 mmol/mol (6.5%) would correspond to different FPG levels in each setting, as shown by a discrepancy of up to 7.8 mmol/l. Such a substantial difference hampers potential strategies for expanding diabetes diagnosis and public health planning in high‐altitude settings, and therefore FPG and the oral glucose tolerance test should be used as diagnostic criteria under these circumstances.

## Funding sources

The CRONICAS Cohort Study was funded in whole with Federal funds from the United States National Heart, Lung, and Blood Institute, National Institutes of Health, Department of Health and Human Services, under contract No. HHSN268200900033C. The PERU MIGRANT Study was funded by the Wellcome Trust (GR074833MA) and Universidad Peruana Cayetano Heredia (Fondo Concursable No. 20205071009). William Checkley was further supported by a Pathway to Independence Award (R00HL096955) from the National Heart, Lung and Blood Institute. AB‐O (103994/Z/14/Z) and LS (098504/Z/12/Z) are both funded by Wellcome Trust. JJM is also supported by Fogarty International Centre (R21TW009982), Grand Challenges Canada (0335‐04), International Development Research Center Canada (106887‐001), Inter‐American Institute for Global Change Research (IAI CRN3036), Medical Research Council UK (M007405), National Heart, Lung, and Blood Institute (U01HL114180), National Institutes of Mental Health (U19MH098780).

## Competing interests

None declared.

## Supporting information


**Table S1.** Linear, quadratic and cubic regression models for HbA_1c_ using glucose‐like predictor (crude and adjusted models).
**Table S2.** Distribution of diabetes and prediabetes at sea level and high altitude considering HbA_1c_ or FPG standard cut‐off points and including cases of diabetes diagnosed by physician and pharmacological treatment.
**Figure S1.** Comparison between ROC curves at sea level (blue) and high altitude (red), for HbA_1c_ standard cut‐off points using FPG as the gold standard of diabetes.
**Figure S2.** Comparison between ROC curves at sea level (blue) and high altitude (red), for HbA_1c_ standard cut‐off points using FPG as the gold standard of prediabetes.
