# Supplementary material for: Glycated haemoglobin (HbA1c) and fasting plasma glucose relationships in sea‐level and high‐altitude settings
Source: Diabet Med. 2017 May 15;34(6):804–12. doi: 10.1111/dme.13335 (PMC5432378; doi:10.1111/dme.13335)
Supplement: Supplementary file 1 — Table S1. Linear, quadratic and cubic regression models for HbA1c using glucose‐like predictor (crude and adjusted models). Table S2. Distribution of diabetes and prediabetes at sea level and high altitude considering HbA1c or FPG standard cut‐off points and including cases of diabetes diagnosed by physician and pharmacological treatment. Figure S1. Comparison between ROC curves at sea level (blue) and high altitude (red), for HbA1c standard cut‐off points using FPG as the gold standard of diabetes. Figure S2. Comparison between ROC curves at sea level (blue) and high altitude (red), for HbA1c standard cut‐off points using FPG as the gold standard of prediabetes. [file DME-34-804-s001.docx]

| **Table S1** Linear, quadratic and cubic regression models for HbA_1c_ using glucose-like predictor (crude and adjusted models) | | | | | | | | | |
| --- | --- | --- | --- | --- | --- | --- | --- | --- | --- |
| Model | Altitude | *n* | Glucose | Glucose ^2 | Glucose ^3 | *P* 1 | *P* 2 | *P* 3 | BIC |
| Linear (Crude) | Sea level | 1930 | 0.0287 | * | * | <0.001 | * | * | 2874.4 |
|  | High altitude | 1215 | 0.0113 | * | * | 0.001 | * | * | 1489.2 |
| Quadratic (Crude) | Sea level | 1930 | 0.0123 | 4.6300E-05 | * | 0.001 | <0.001 | * | 2791.7 |
|  | High altitude | 1215 | 0.0101 | 4.5500E-06 | * | 0.25 | 0.91 | * | 1496.0 |
| Cubic (Crude) | Sea level | 1930 | -0.0201 | 0.0003 | -3.87E-07 | 0.22 | 0.03 | 0.08 | 2767.1 |
|  | High altitude | 1215 | 0.0374 | -0.0002 | 4.13E-07 | 0.21 | 0.40 | 0.42 | 1493.4 |
| Linear (Adjusted) | Sea level | 1928 | 0.0286 | * | * | <0.001 | * | * | 2787.9 |
|  | High altitude | ***1209*** | ***0.0099*** | ******* | ******* | ***0.01*** | ******* | ******* | ***1356.5*** |
| Quadratic (Adjusted) | Sea level | ***1928*** | ***0.0097*** | ***0.0001*** | ******* | ***0.01*** | ***<0.001*** | ******* | ***2678.9*** |
|  | High altitude | 1209 | 0.0053 | 1.6700E-05 | * | 0.55 | 0.68 | * | 1358.7 |
| Cubic (Adjusted) | Sea level | 1928 | -0.0249 | 0.0003 | -4.08E-07 | 0.13 | 0.02 | 0.06 | 2647.9 |
|  | High altitude | 1209 | 0.0284 | -0.0002 | -3.46E-07 | 0.34 | 0.52 | 0.50 | 1358.1 |
| Betas were estimated by maximum likelihood (Newton-Raphson) and P-values were performed using robust variance estimates. | | | | | | | | | |
| Glucose ^3 corresponds to regression coefficient of cubed glucose, Glucose ^2 corresponds to regression coefficient of squared glucose and Glucose correspond to regression coefficient of a simple Glucose measure. | | | | | | | | | |
| The differences between sample sizes of crude and adjusted models were minimal: *n* at sea level was 1930 (crude) compared with 1928 (adjusted model), and corresponding figures at high altitude were 1215 and 1209, respectively. Values were missing at random. | | | | | | | | | |
| Models were adjusted by age, sex, education, wealth, BMI and haemoglobin. | | | | | | | | | |
| Models in bold and italic are final selected models. | | | | | | | | | |

# Table S2 Distribution of diabetes and prediabetes at sea level and high altitude considering HbA_1c_ or FPG standard cut-off points and including cases of diabetes diagnosed by physician and pharmacological treatment

|  |  | Sea level | | High altitude | |
| --- | --- | --- | --- | --- | --- |
| Test | Dx | % | *n** | % | *n*^*^ |
| HbA_1c_ | Normal | 40.9 | 789 | 36.0 | 438 |
|  | Prediabetes | 50.7 | 978 | 58.3 | 709 |
|  | Diabetes | 8.4 | 163 | 5.7 | 69 |
|  | *Total* | *100.0* | *1930* | *100.0* | *1216* |
| FPG | Normal | 74.2 | 1433 | 87.2 | 1060 |
|  | Prediabetes | 22.5 | 434 | 11.0 | 134 |
|  | Diabetes | 3.3 | 63 | 1.8 | 22 |
|  | *Total* | *100.0* | *1930* | *100.0* | *1216* |
| Diagnostic criteria for HbA_1c_: diabetes HbA_1c_ ≥ 6.5%; prediabetes 6.5% > HbA_1c_ ≥ 5.7%; normal HbA_1c_ < 5.7%. | | | | | |
| Diagnostic criteria for FPG: diabetes FPG ≥ 126 mg/dl; prediabetes 126 > FPG ≥ 100 mg/dl; normal FPG < 100 mg/dl.  *Of the 3613 people in the study, we excluded those without complete data to evaluate diabetes status (HbA_1c_ or FPG, n=467), therefore data from *n* = 3145 individuals are included in this table. | | | | | |

# Figure S1 Comparison between ROC curves at sea level (blue) and high altitude (red), for HbA_1c_ standard cut-off points using FPG as the gold standard of diabetes.

# Figure S2 Comparison between ROC curves at sea level (blue) and high altitude (red), for HbA_1c_ standard cut-off points using FPG as the gold standard of prediabetes.
